# Supplementary material for: TISMorph: A tool to quantify texture, irregularity and spreading of single cells
Source: PLoS One. 2019 Jun 3;14(6):e0217346. doi: 10.1371/journal.pone.0217346 (PMC6546208; doi:10.1371/journal.pone.0217346)
Supplement: S1 File — (PDF) [file pone.0217346.s001.pdf]

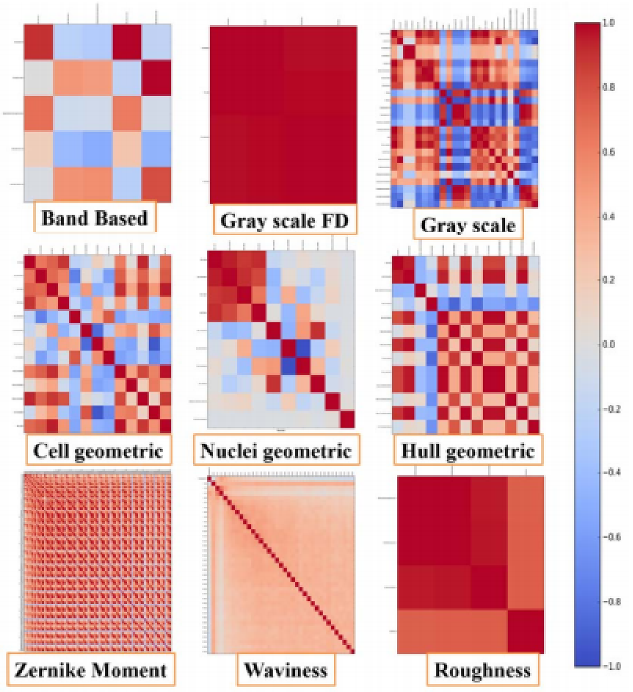

Figure A: Pearson correlation coefficient between features within each shape category. Within each shape category features are highly correlated other than band based, waviness (Fourier), and some of the nuclei measures. Each pixel is the average of Pearson correlation for 14 cases, 7 drugs x 2 cell lines.

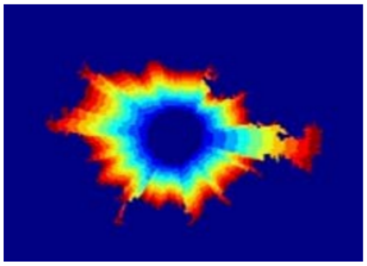

Figure B: Band based division of textural image of a cell. Cell is divided into 10 equally spaced radial bands. All the measures calculated based on this representation is listed in Table B.

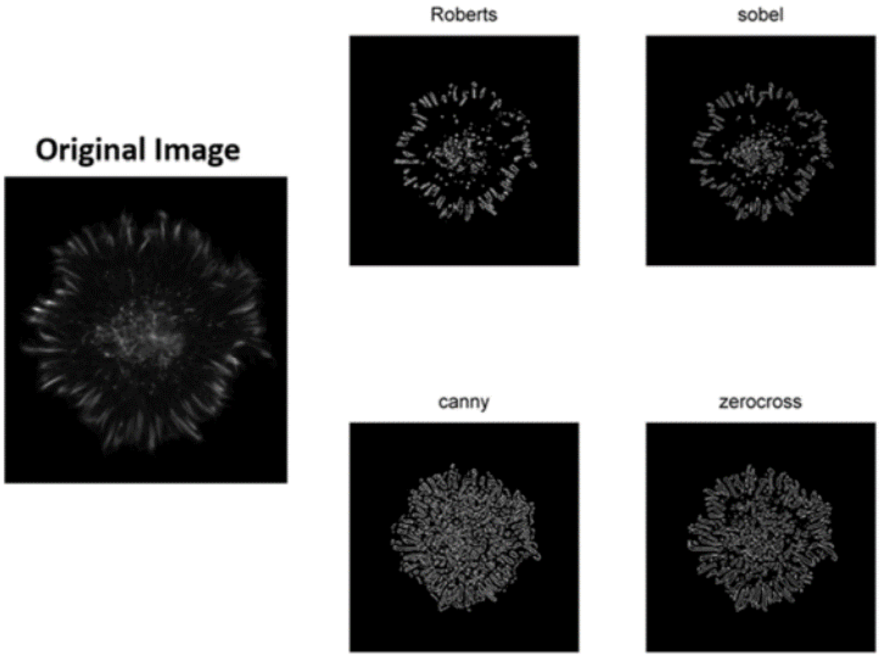

Figure C: Matlab Edging methods used to binerize gray scale image of actin. Four different methods of Roberts, Sobel, Canny, and zerocross are used in this paper.

(a) (b)

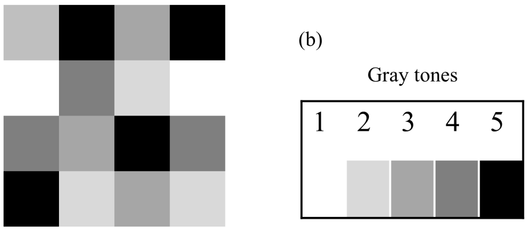

(c)

2	5	3	5
1	4	2	1
4	3	5	4
5	2	3	2

(d)

Gray tone of : origin ↓,destination →	1	2	3	4	5
1	(1,1)	(1,2)	(1,3)	(1,4)	(1,5)
2	(2,1)	(2,2)	(2,3)	(2,4)	(2,5)
3	(3,1)	(3,2)	(3,3)	(3,4)	(3,5)
4	(4,1)	(4,2)	(4,3)	(4,4)	(4,5)
5	(5,1)	(5,2)	(5,3)	(5,4)	(5,5)

Figure D: Calculation of GCLM matrix. a) A cartoon of a 4pixels x 4pixels image. b) As shown in this figure the image has 5 gray tones. c) Numerical representation of image based on gray tone is shown in this figure. d) GCLM Matrix is 5 x 5 element and its ij^{th} element is number of the times which pixel with intensity of j is in distance $\vec{d}$ from the pixels with intensity of i. The vector $\vec{d}$ is an arbitrary vector but should be defined in advance. In this paper $\vec{d}$ is defined to have length of one and it is defined in 4 directions of 0°, 45°, 90°, and 135°. Gray scale measures are calculated based on four directions and their average is reported in this paper.

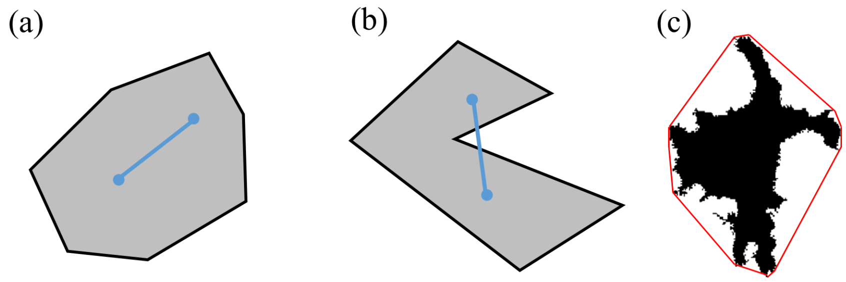

Figure E: Convex Hull. A cartoon of a) a convex and b) a non-convex polygon. A convex polygon is a polygon which any line connecting two points inside the polygon falls inside the polygon. c. Convex hull surrounding a cell.

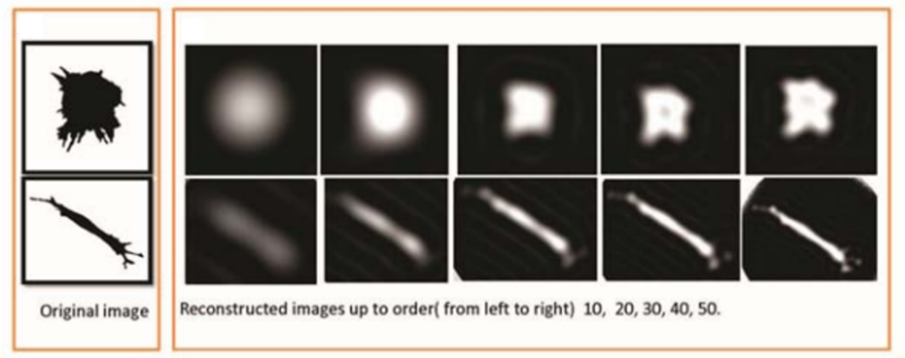

Figure F: Reconstruction of cell images for two different shaped cells. Left, original images. Right, Reconstructed images using Zernike moments up to order (from left to the right) 10, 20, 30, 40, 50.[1]

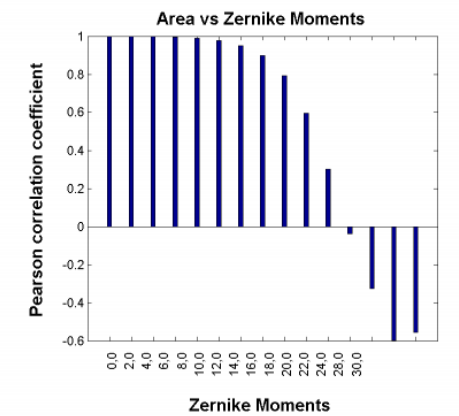

Figure G: Pearson correlation coefficient between Zernike Moments with $m = 0$ and Cell Area. Correlation is maximum and positive with Zernike moment of 0-0, it decreases with increasing the order and it is almost 0 for Zernike moment of 22-0 and it is negative for higher orders and increases in magnitude. Each data point is the average of Pearson correlation for 14 cases, 7 drugs x 2 cell lines.

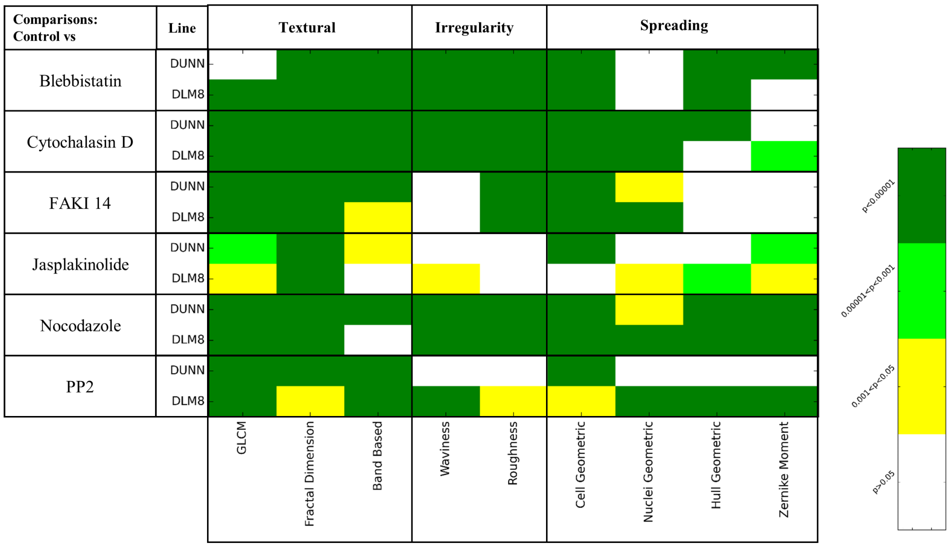

Figure H: Comparisons of means using t-tests. The figure shows a heat map of the p values obtained by comparisons of control cells with the experimental conditions shown for each major category of shape features.

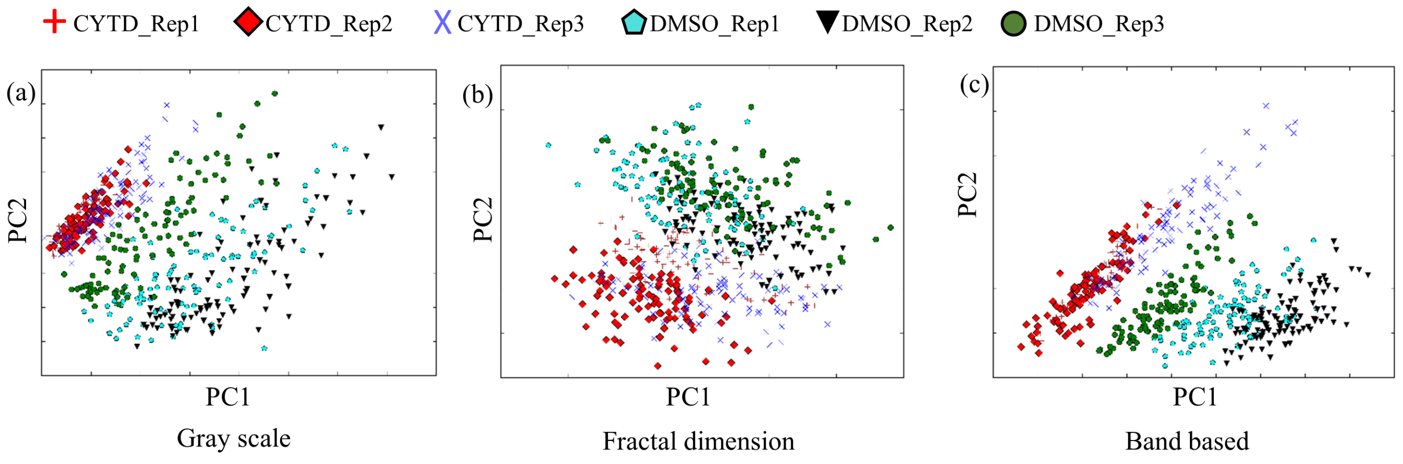

Figure I: Reproducibility of Technical Replicates. The morphometric feature categories of three technical replicates of cells treated with Cytochalasin D and controls (DMSO treated) are compared. The figure shows that for all three categories that were developed for this work, morphometric data from technical replicates of the same conditions overlap with each other.

Table A: Description of the drugs used to perturb cytoskeleton.

Drug	Blebbistatin	Cytochalasin D	Jasplakinolide	FAKI 14	Nocodazole	PP2
Target	Inhibits non-muscle myosin II activity	Inhibits actin polymerization	1.Promotes actin polymerization 2.Stabilizes actin filaments	Inhibits focal adhesion kinase	Depolymerizing microtubules	Inhibits Src kinase
Application in cancer therapy	Inhibits cancer invasion [2]	Used as chemotherapeutic agent [3, 4].	Reduces lung metastases of systemic Lewis lung carcinoma[5]).	Showed anti-metastatic, anti-neoplastic and anti-angiogenic properties [6, 7, 8, 9].	Has chemotherapeutic properties by arresting cells in mitosis[10]	Prevention of cancer metastasis[11].
Effect	1.Reduces F-actin contractility 2.Increase in cell spreading and 3.Decreases cell motility[12]	Decreases cell contractility	Stabilizes F-actin [13]	Reduces cell motility and focal adhesion turnover[14].	Cell shapes which lack tails and long protrusions and decreased cell asymmetry[15].	Induces strong cell-cell contact and Enhances E-cadherin/catenin expression which are strongly associated with the actin cytoskeleton[11].
Concentration (μM)	10	1	0.01	1	5	1
Vendor	Santa Cruz Biotechnology	Calbiochem	Calbiochem	Santa Cruz Biotechnology	Sigma Alrdich	Calbiochem

Table B: Band based measurements used in the paper [16]

Parameter	Descriptions	Formula
Max band intensity	Intensity of the band with maximum average intensity	Max $(I_{B_1}, \ldots, I_{B_{10}})$
Max band index	Location of the band with maximum average intensity	The n such that $I_n =$ Max $(I_{B_1}, \ldots, I_{B_{10}})$
Min band intensity	Intensity of the band with minimum average intensity	Min $(I_{B_1}, \ldots, I_{B_{10}})$
Min band index	Location of the band with minimum average intensity	The m such that $I_m =$ Min $(I_{B_1}, \ldots, I_{B_{10}})$
Above average adjusted intensity	Adjusted, weighted sum of average actin intensities greater than the mean intensity.	$\frac{\sum_{k=1}^{10} s(I_{B_k})(I_{B_k})}{Max(I_{B_1},\ldots,I_{B_{10}}) \sum_{k=1}^{10} s(I_{B_k})}$ where $s(I) = \begin{cases} 1, & \text{if } I \geq mean(I_{B_1} \ldots I_{B_{10}}) \\ 0, & \text{otherwise} \end{cases}$

Table C: Gray scale measures used in this paper. See Table D for the parameters used in this table.

Parameter	Formula
Autocorrelation	$\sum_{i=1}^{N_g}\sum_{j=1}^{N_g} ijp(i,j)$
Contrast M(Matlab)	$\sum_{i=1}^{N_g}\sum_{j=1}^{N_g} \|i-j\|^2 p(i,j)$
Correlation M(Matlab)	$\sum_{i=1}^{N_g}\sum_{j=1}^{N_g} \frac{(i-\mu i)(j-\mu j)p(i,j)}{\sigma_i\sigma_j}$
Correlation P	$\sum_{i=1}^{N_g}\sum_{j=1}^{N_g} \frac{ijp(i,j)-\mu_x\mu_y}{\sigma_i\sigma_j}$
Cluster Standard deviation	$\sum_{i=1}^{N_g}\sum_{j=1}^{N_g} (i+j-\mu_x-\mu y)^2 p(i,j)$
Cluster Prominence	$\sum_{i=1}^{N_g}\sum_{j=1}^{N_g} (i+j-\mu_x-\mu y)^4 p(i,j)$
Cluster Shade	$\sum_{i=1}^{N_g}\sum_{j=1}^{N_g} (i+j-\mu_x-\mu y)^3 p(i,j)$
Dissimilarity	$\sum_{i=1}^{N_g}\sum_{j=1}^{N_g} \|i-j\|p(i,j)$
Energy	$\sum_{i=1}^{N_g}\sum_{j=1}^{N_g} p^2(i,j)$
Entropy	-$\sum_{i=1}^{N_g}\sum_{j=1}^{N_g} p(i,j)\log(p(i,j)$
Homogeneity M(Matlab)	$\sum_{i=1}^{N_g}\sum_{j=1}^{N_g} \frac{p(i,j)}{1+\|i-j\|}$
Homogeneity P	$\sum_{i=1}^{N_g}\sum_{j=1}^{N_g} \frac{p(i,j)}{1+(i-j)^2}$
Maximum probability	$MAX_{i,j}p(i,j)$
Sum squares variance	$\sum_{i=1}^{N_g}\sum_{j=1}^{N_g} (i-\mu)^2 p(i,j)$
Sum average	$\sum_{i=2}^{2N_g} ip_{x+y}(i)$
Sum variance	$\sum_{i=2}^{2N_g} (i-SumEntropy)^2 p_{x+y}(i)$
Sum Entropy	-$\sum_{i=2}^{2N_g} p_{x+y}(i)\log\left(p_{x+y}(i)\right)$
Difference Variance	$\sum_{i=0}^{N_g-1} i^2 p_{x-y}(i)$
Difference Entropy	-$\sum_{i=0}^{N_g-1} p_{x-y}(i)\log\left(p_{x-y}(i)\right)$
Information Measure of Correlation1	$\frac{Entropy-HXY1}{(max(HX,HY)}$
Information measure Correlation 2	$\sqrt{1-e^{-2(HXY2-HXY)}}$
Inverse Difference Normalized(INN) (Matlab)	$\sum_{i=1}^{N_g}\sum_{j=1}^{N_g} \frac{p(i,j)}{1+\frac{\|i-j\|}{N_g}}$
Inverse Difference Moment Normalized(Matlab)	$\sum_{i=1}^{N_g}\sum_{j=1}^{N_g} \frac{p(i,j)}{1+\frac{(i-j)^2}{N_g^2}}$

Table D: Parameters used in the definitions of gray scale measures in the Table C.

Parameter	Formula
$P(i,j)$	ij^{th} entry of co-occurance probability matrix
N_g	Highest intensity of gray level
μ	Mean value of $p(i,j)$
$p_x(i)$	$\sum_{j=1}^{N_g} p(i,j)$
$p_y(i)$	$\sum_{i=1}^{N_g} p(i,j)$
μ_x	$\sum_{i=1}^{N_g} \sum_{j=1}^{N_g} ip(i,j)$
μ_y	$\sum_{i=1}^{N_g} \sum_{j=1}^{N_g} jp(i,j)$
σ_x^2	$\sum_{i=1}^{N_g} \sum_{j=1}^{N_g} (i-\mu_x)^2 p(i,j)$
σ_y^2	$\sum_{i=1}^{N_g} \sum_{j=1}^{N_g} (j-\mu_y)^2 p(i,j)$
$p_{x+y}(k)$	$\sum_{i=1}^{N_g} \sum_{j=1}^{N_g} p(i,j)$ where $i+j=k, k=2,..,2N_g$
$p_{x-y}(k)$	$\sum_{i=1}^{N_g} \sum_{j=1}^{N_g} p(i,j)$ where $i-j=k, k=0,..,N_g-1$
HX	-$\sum_{i=1}^{N_g} p_x(i) \log p_x(i)$
HY	-$\sum_{i=1}^{N_g} p_y(i) \log p_y(i)$
$HXY1$	-$\sum_{i=1}^{N_g} \sum_{j=1}^{N_g} p(i,j) \log (p_x(i)p_y(j))$
$HXY2$	-$\sum_{i=1}^{N_g} \sum_{j=1}^{N_g} p_x(i)p_y(j) \log (p_x(i)p_y(j))$

Table E: Geometric parameters.

Parameter	Description	Unit
Cell area	Area of the cell	$Pixel^2$
Cell perimeter	Perimeter of the cell	$Pixel$
Cell major axis	The major axis of an ellipse drawn around the cell	$Pixel$
Cell minor axis	The minor axis of an ellipse drawn around the cell	$Pixel$
Cell circularity	$\frac{4Area}{Perimeter^2}$	$Unit - less$
Cell aspect Ratio	$\frac{Cell\ Major\ axis}{Cell\ Minor\ axis}$	$Unit - less$
Cell roundness	$\frac{4\pi Area}{\pi\ \ Cell\ Major\ axis^2}$	$Unit - less$
Cell solidity	$\frac{Convex\ hull\ area}{Cell\ area}$	$Unit - less$
Max cell Radius	Maximum radius of the cell	$Pixel$
Cell radius ratio	$\frac{Max\ radius\ of\ the\ cell}{Min\ radius\ of\ the\ cell}$	$Unit - less$
Mean Cell Radius	The mean of all radii drawn from the hull's centroid to an exterior point	$Pixel$
CV Cell Radius	The relative variation of radii drawn from the cells center to an exterior point. Given by the Standard of Deviation of all Radii divided by the mean of all radii	$Unit - less$
Max Span	The maximum distance from one point on the convex hull to another	$Pixel$

Table F: Nuclei geometric parameters.

Parameter	Description	Unit
Nuc area	Area of the nucleus	$Pixel^2$
Nuc perimeter	Perimeter of the nucleus	$Pixel$
Nuc major	Major axis of an ellipse drawn around the nucleus	$Pixel$
Nuc minor	Minor axis of an ellipse drawn around the nucleus	$Pixel$
Nuc circularity	$\frac{4\pi Nuc\, area}{Nuc\, perimeter^2}$	$Unit - less$
Nuc aspect ratio	$\frac{Nuc\, major}{Nuc\, minor}$	$Unit - less$
Nuc roundness	$\frac{4\pi Nuc\, area}{\pi Nuc\, major^2}$	$Unit - less$
Nuc solidity	$\frac{Area\, of\, convex\, hull\, of\, nuclei)}{Nuc\, area}$	$Unit - less$
Distance nuc to cell centroid	Distance between the centroids of cell and nucleus	$Pixel$
Nuc to cell orientation	Angle between the distance nuc to cell centroid and cell major axis	$Degree$

Table G: Convex hull geometric parameters.

Parameter	Description	Unit
Hull area	Area of the convex hull	$Pixel^2$
Hull perimeter	Perimeter of the convex hull.	$Pixel$
Ratio of perimeter of Hull to cell	Ratio of hull perimeter to cell perimeter.	$Unit - less$
Hull circularity	$\frac{4\pi\ Hull\ area}{Hull\ perimeter^2}$	$Unit - less$
Max hull Radius	Maximum distance from centroid of Hull to an exterior point on the hull.	$Pixel$
Hull radius ratio	Maximum/minimum radius of hull.	$Unit - less$
Mean hull radius	The mean of all radii drawn from the hull's centroid to an exterior point.	$Pixel$
CV hull radius	(STD hull radius)/(Mean hull radius)	$Unit - less$
Bounding circle diameter	The diameter of the bounding circle drawn around the cell.	$Pixel$
Max circle to hull radius	The maximum distance from the center of the bounding circle to an edge of the convex hull.	$Pixel$
Circle to hull radius ratio	(Max circle to hull radius)/(Min circle to hull radius)	$Unit - less$
Mean circle to hull radius	The mean of all radii drawn from the circle's center to the hull	$Pixel$
CV Circle2Hull Radius	(STD(all radii drawn from the circle s center to the hull))/(Mean circle to hull radius)	$Unit - less$

Table H: Roughness measurements.

Parameter	Formula	Units
Mean of absolute values	$R_a = \frac{1}{n}\sum_{i=1}^{n} \lvert y_i \rvert$	*Pixel*
Root mean squared	$R_{rms} = \sqrt{\frac{1}{n}\sum_{i=1}^{n} y_i^2}$	*Pixel*
Maximum height of the profile	$R_{yt} = R_p - R_v$ Where R_p has the maximum and R_v has the minimum height in the profile	*Pixel*
Kurtosis	$R_{Ku} = \frac{1}{nR_{rms}^4}\sum_{i=1}^{n} y_i^4$	$Pixel^4$

Table I: Significance of changes in primary principal component (PPC) of morphometric classes for different drugs according to T-test analysis. The p-value of the null hypothesis that the experimental condition has the same mean as the control is reported for each class of morphometrics, based on the PPC. There are significant changes (p-value $< 1E-3$) in some actin textural measures (Band-Based, Fractal Dimension and Gray scale measures), spreading measures (Cell Geometric, Nuclei Geometric, Hull Geometric and Zernike moment) and Irregularity measures (Waviness and Roughness) for every experimental condition. Sample size is 300 cells for each cell line and drug condition other than DLM8 FAKI 14 which has a sample size of 200.

		Textural			Spreading				Irregularity	
Comparisons: Control vs	Cell Line	Band Based	Fractal Dimension	Gray Scale	Cell Geometric	Nuclei	Hull Geometric	Zernike Moment	Waviness	Roughness
Blebbistatin	DUNN	1.00E-07	2.60E-14	0.008	1.60E-78	0.006	3.10E-14	3.00E-21	2.00E-35	2.70E-45
	DLM8	3.00E-08	6.30E-45	6.00E-09	6.00E-25	0.004	7.90E-08	0.0018	2.00E-12	5.30E-16
Cytochalasin D	DUNN	3.00E-50	2.10E-31	2.00E-23	3.50E-21	2.00E-22	2.50E-06	0.0092	5.00E-10	3.30E-27
	DLM8	4.00E-31	2.10E-13	1.00E-23	4.50E-15	1.00E-18	0.05285	1.00E-05	3.00E-07	8.20E-07
FAKI 14	DUNN	2.00E-09	5.16E-31	1.00E-23	5.20E-09	2.00E-04	0.04184	0.1194	0.1398	2.20E-12
	DLM8	4.00E-04	6.60E-22	2.00E-21	1.50E-06	2.00E-06	0.07864	0.0997	0.3125	7.80E-07
Jasplakinolide	DUNN	2.00E-04	2.10E-13	9.00E-05	1.30E-08	0.001	0.02999	4.00E-05	0.0036	0.051
	DLM8	0.003	2.80E-09	2.00E-04	0.00207	5.00E-04	1.20E-05	0.0005	0.0001	0.02117
Nocodazole	DUNN	6.00E-13	5.60E-31	3.00E-25	1.30E-28	1.00E-04	4.60E-10	9.00E-07	1.00E-12	3.30E-16
	DLM8	0.001	9.80E-14	4.00E-07	1.20E-14	6.00E-06	3.00E-09	6.00E-14	3.00E-21	5.30E-13
PP2	DUNN	2.00E-21	1.40E-11	2.00E-10	2.50E-06	0.003	0.03602	0.0243	0.0089	0.00387
	DLM8	4.00E-07	0.00035	3.00E-09	0.00043	8.00E-10	2.70E-09	4.00E-13	4.00E-11	0.00078

References

[1] Alizadeh E, Lyons SM, Castle JM, Prasad A. Measuring systematic changes in invasive cancer cell shape using Zernike moments. Integr Biol. 2016;8(11):1183–1193. doi:10.1039/C6IB00100A.

[2] Nelson T, Roddy M, Appledorn DM, Groppi V. The myosin II inhibitor blebbistatin more potently inhibits 3-D cell invasion than 2-D cell migration in a human metastatic tumor cell. Cancer Research. 2011;71(18 Supplement):C1—-C1. doi:10.1158/1538-7445.FBCR11-C1.

[3] Trendowski M, Zoino JN, Christen TD, Acquafondata C, Fondy TP. Preparation, In Vivo Administration, Dose-Limiting Toxicities, and Antineoplastic Activity of Cytochalasin B. Translational Oncology. 2015;8(4):308–317. doi:10.1016/j.tranon.2015.06.003.

[4] Trendowski M, Mitchell JM, Corsette CM, Acquafondata C, Fondy TP. Chemotherapy with cytochalasin congeners in vitro and in vivo against murine models. Investigational New Drugs. 2015;33(2):290–299. doi:10.1007/s10637-014-0203-5.

[5] Takeuchi H, Ara G, Sausville EA, Teicher B. Jasplakinolide: interaction with radiation and hyperthermia in human prostate carcinoma and Lewis lung carcinoma. Cancer chemotherapy and pharmacology. 1998;42(6):491–496.

[6] Kang CG, Han HJ, Lee HJ, Kim SH, Lee EO. Rho-associated kinase signaling is required for osteopontin-induced cell invasion through inactivating cofilin in human non-small cell lung cancer cell lines. Bioorganic & medicinal chemistry letters. 2015;25(9):1956–60. doi:10.1016/j.bmcl.2015.03.024.

[7] Gabarra-Niecko V, Schaller MD, Dunty JM. FAK regulates biological processes important for the pathogenesis of cancer. Cancer metastasis reviews. 2003;22(4):359–74.

[8] McLean GW, Avizienyte E, Frame MC. Focal adhesion kinase as a potential target in oncology. Expert opinion on pharmacotherapy. 2003;4(2):227–34. doi:10.1517/14656566.4.2.227.

[9] Hecker TP, Gladson CL. Focal adhesion kinase in cancer. Frontiers in bioscience : a journal and virtual library. 2003;8:s705–14.

[10] Endo K, Mizuguchi M, Harata A, Itoh G, Tanaka K. Nocodazole induces mitotic cell death with apoptotic-like features in Saccharomyces cerevisiae. FEBS Letters. 2010;584(11):2387–2392. doi:https://doi.org/10.1016/j.febslet.2010.04.029.

[11] Nam Js, Ino Y, Sakamoto M, Hirohashi S. Src family kinase inhibitor PP2 restores the E-cadherin/catenin cell adhesion system in human cancer cells

and reduces cancer metastasis. Clinical Cancer Research. 2002;8(7):2430–6. doi:do.

[12] Sero JE, Sailem HZ, Ardy RC, Almuttaqi H, Zhang T, Bakal C. Cell shape and the microenvironment regulate nuclear translocation of NF-κB in breast epithelial and tumor cells. Molecular systems biology. 2015;11(3):790. doi:10.15252/msb.20145644.

[13] Deposit AC, Transfer AM, Transfer W, Deposit C, Pay CQ, Card C, et al. No Title; p. 1–4.

[14] Schlaepfer DD, Hauck CR, Sieg DJ. Signaling through focal adhesion kinase. Progress in biophysics and molecular biology. 1999;71(3-4):435–78.

[15] Rodionov VI, Gyoeva FK, Tanaka E, Bershadsky AD, Vasiliev JM, Gelfand VI. Microtubule-dependent control of cell shape and pseudopodial activity is inhibited by the antibody to kinesin motor domain. The Journal of Cell Biology. 1993;123(6).

[16] Slater JH, Culver JC, Long BL, Hu CW, Hu J, Birk TF, et al. Recapitulation and Modulation of the Cellular Architecture of a User-Chosen Cell of Interest Using Cell-Derived, Biomimetic Patterning. ACS Nano. 2015;9(6):6128–6138. doi:10.1021/acsnano.5b01366.
